# Achieving the critical view of safety in the difficult laparoscopic cholecystectomy: a prospective study of predictors of failure

**DOI:** 10.1007/s00464-020-08093-3

**Published:** 2020-10-16

**Authors:** Ahmad H. M. Nassar, Hwei J. Ng, Arkadiusz Peter Wysocki, Khurram Shahzad Khan, Ines C. Gil

**Affiliations:** 1 University Hospital Monklands, Airdrie, Lanarkshire, ML6 0JSb Scotland; 2 NHS Greater Glasgow and Clyde, Glasgow, UK; 3 Logan Hospital, Corner Meadowbrook and Loganlea Roads, Meadowbrook, Logan City, QLD 4133 Australia; 4 Centro Hospitalar de Leiria, Leiria, Portugal

**Keywords:** Laparoscopic cholecystectomy, Salvage cholecystectomy, Critical view of safety, Difficulty grading, Nassar difficulty scale, Bile duct injury, Cholecystectomy complications, Subtotal cholecystectomy, Fundus first dissection

## Abstract

**Background:**

Bile duct injury rates for laparoscopic cholecystectomy (LC) remain higher than during open cholecystectomy. The “culture of safety” concept is based on demonstrating the critical view of safety (CVS) and/or correctly interpreting intraoperative cholangiography (IOC). However, the CVS may not always be achievable due to difficult anatomy or pathology. Safety may be enhanced if surgeons assess difficulties objectively, recognise instances where a CVS is unachievable and be familiar with recovery strategies.

**Aims and methods:**

A prospective study was conducted to evaluate the achievability of the CVS during all consecutive LC performed over four years. The primary aim was to study the association between the inability to obtain the CVS and an objective measure of operative difficulty. The secondary aim was to identify preoperative and operative predictors indicating the use of alternate strategies to complete the operation safely.

**Results:**

The study included 1060 consecutive LC. The median age was 53 years, male to female ratio was 1:2.1 and 54.9% were emergency admissions. CVS was obtained in 84.2%, the majority being difficulty grade I or II (70.7%). Displaying the CVS failed in 167 LC (15.8%): including 55.6% of all difficulty grade IV LC and 92.3% of difficulty grade V. There were no biliary injuries or conversions.

**Conclusion:**

All three components of the critical view of safety could not be demonstrated in one out of 6 consecutive laparoscopic cholecystectomies. Preoperative factors and operative difficulty grading can predict cases where the CVS may not be achievable. Adapting instrument selection and alternate dissection strategies would then need to be considered.

The incidence of major bile duct injury (BDI) during laparoscopic cholecystectomy (LC) has remained relatively constant ranging from 0.1% to 1.5% [1, 2] despite improvements in equipment and techniques, while the incidence during open cholecystectomy is typically quoted as 0.2% [3]. Avoiding bile duct injury is important as this results in additional morbidity, mortality and escalation of health care costs [4].

Strasberg et al. suggested that one of the main causes of BDI is the misidentification of the bile duct as the cystic duct or artery [5, 6] and described the ‘critical view of safety’ (CVS) in 1995 [7]. It is widely adopted and taught as a method of target identification during LC but two decades since its introduction there does not seem to be a reduction in biliary injuries [6]. CVS is included in the Safe Cholecystectomy Programme published by the Society of American Gastrointestinal and Endoscopic Surgeons (SAGES) as one of the six strategies recommended to help minimise bile duct injuries [8]. The three elements of CVS are: gallbladder hilum being free of connective tissue, only two structures entering the gallbladder and at least one third of the gallbladder mobilised off the cystic plate. However, as many as one-third of patients will have three structures in Calot’s triangle [9] and pathologies such as a fibrotic cystic pedicle or cholecysto-choledochal fistula may mean the CVS is not achievable.

Multiple strategies have been recommended for when the CVS is impossible to display [10] but no objective definition of the difficulty of cholecystectomy was used in these recommendations. Our study was designed to evaluate the frequency of inability to obtain the CVS, to explore preoperative predictive factors that may warn the surgeon of the potential for CVS failure and to evaluate the association with an established objective operative difficulty grading system.

## Methods

A prospective study of consecutive laparoscopic cholecystectomies performed between January 2016 and December 2019 was conducted to specifically assess issues relating to the CVS and the feasibility of displaying it during every LC. This cohort represents the last 5th of the senior surgeon’s experience of 5675 LC over 28 years. This firm is a referral unit subspecialising in biliary emergencies for over 25 years and as such, it deals with a significant percentage of complex cases. No ethical approval was necessary as this was a clinical study using a standard protocol for LC. The procedures were performed by the senior author or by his trainees under direct on table supervision. Data on patient demographics, type of admission, clinical presentation, radiological findings, interval from admission to surgery, operative difficulty grade, achievement of CVS, operative time, conversion to open, perioperative complications, re-admissions and mortality were recorded. The operative difficulty grade was based on the Nassar Scale [11] (Table 1). This scale was validated as a tool of reporting operative findings and technical difficulty in 2 different large datasets including the CholeS study and found to standardise the description of operative findings by multiple grades of surgeons in over 8800 cases [12].

**Table 1:** Operative difficulty grading: modified nassar scale

Grade	Description
I	Gallbladder—floppy, non-adherent
	Cystic pedicle—thin and clear
	Adhesions—simple up to the neck/Hartmann's pouch
II	Gallbladder—mucocele, packed with stones
	Cystic pedicle—fat laden
	Adhesions—simple up to the body
III	Gallbladder—deep fossa, acute cholecystitis, contracted, fibrosis, Hartmann’s adherent to CBD, impaction
	Cystic pedicle—abnormal anatomy or cystic duct—short, dilated or obscured
	Adhesions—dense up to fundus; involving hepatic flexure or duodenum
IV	Gallbladder—completely obscured, empyema, gangrene, mass
	Cystic pedicle—impossible to clarify
	Adhesions—dense, fibrosis, wrapping the gallbladder, duodenum or hepatic flexure difficult to separate
V	Mirizzi Syndrome type 2 or higher, cholecysto-cutaneous, cholecysto-duodenal or cholecysto-colic fistula

This biliary firm managed, by protocol, most referrals of biliary emergencies within the hospital and occasionally inter-hospital transfers. An emergency workload of 60% is agreed according to the senior surgeon’s job plan. The unit adopts single session laparoscopic management of bile duct stones. Endoscopic Retrograde Cholangiopancreatography (ERCP) is not relied upon for preoperative clearance of choledocholithiasis and it is only used in patients unfit for general anaesthesia.

Informed consent was obtained from all patients with specific emphasis on the specialisation of the unit with regard to the management of suspected bile duct stones. IRB approval was not required as the management protocols were consistent with the recommendations of national and international societies.

### Operative technique

Laparoscopic cholecystectomy was performed using a standard four port technique with the patient in the American position. The standard approach in this study was to routinely pursue and display a CVS where possible. The operative difficulty grade was defined as early as possible. Blunt dissection with a “duckbill” forceps was used to clear fat and fibrous tissue over the cystic pedicle, maintaining the dissection lateral to the cystic lymph node (CLN). We do not use the diathermy hook. Once the cystic artery was encircled as it entered the gallbladder wall and the gallbladder neck was positively identified attention was directed to separating the proximal third of the gallbladder from the liver, exposing the cholecystohepatic plate and confirming the presence of a window. Intraoperative cholangiography (IOC) was routinely attempted.

**Fig. 1:**
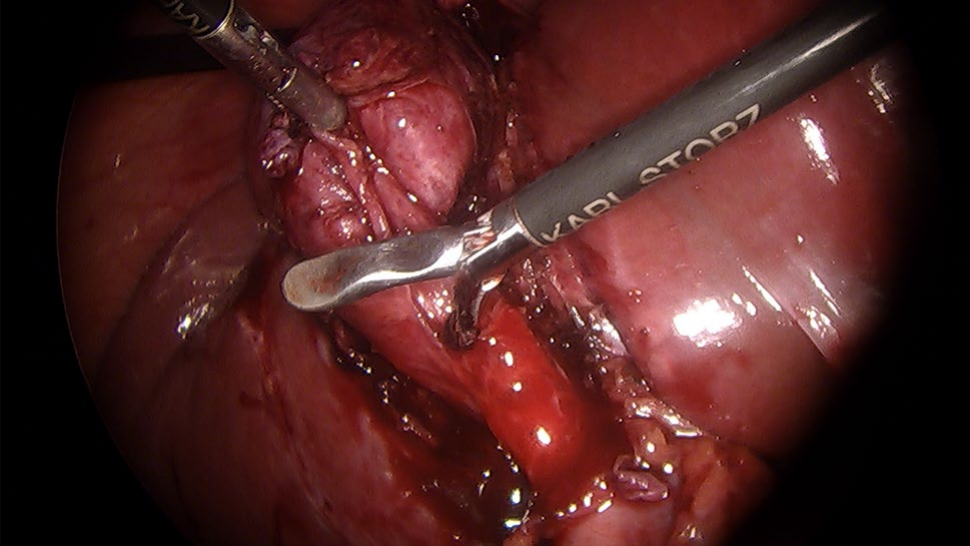
Subserosal dissection of an inflamed, thick-walled gallbladder

**Fig. 2:**
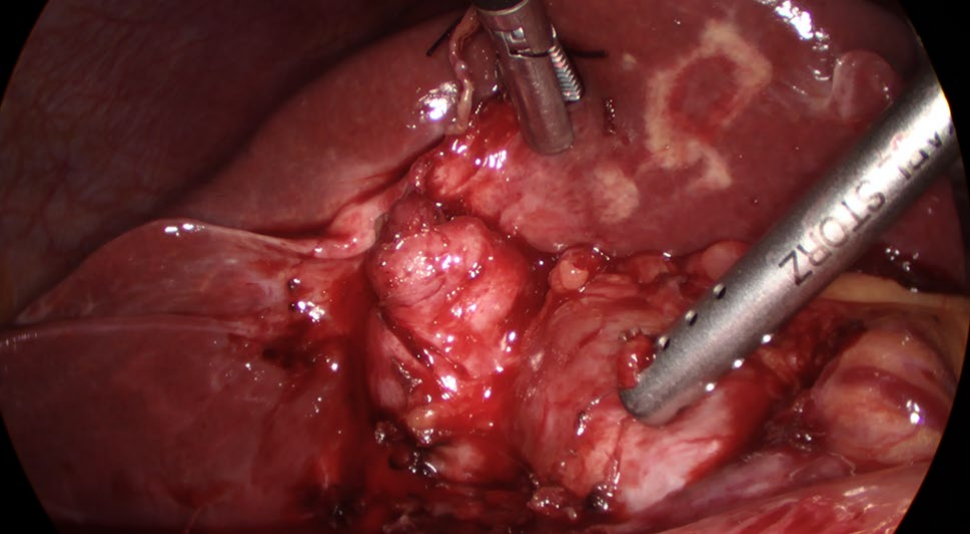
Contracted gallbladder withdrawing a dilated bile duct laterally. CVS was impossible

**Fig. 3:**
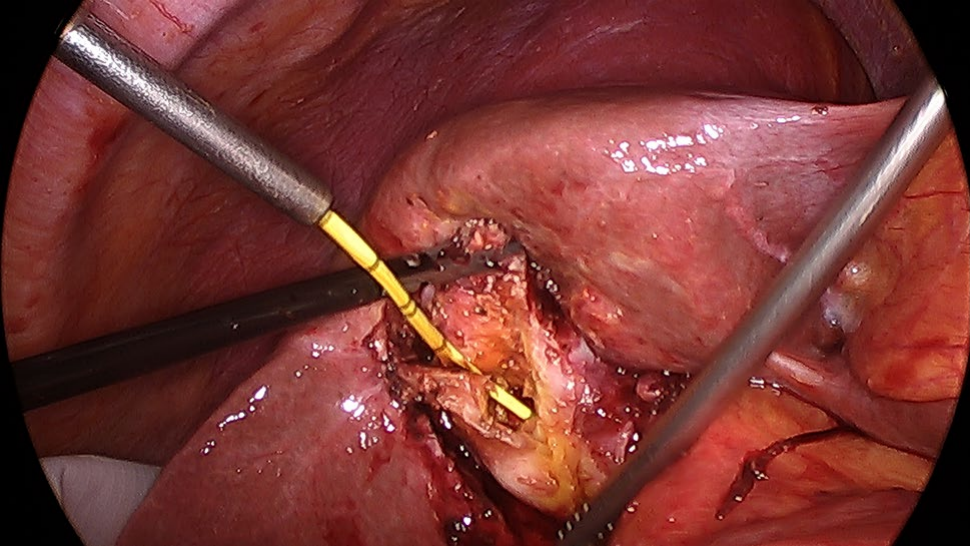
Transvesical cholangiography

When a difficult cholecystectomy was encountered and an area of significant risk was approached, dissection stopped short of any suspected arterial or ductal structures allowing for a time-out pause to consider appropriate strategies. The following difficulty cues and recoveries were noted:

1. A tense gallbladder (acute cholecystitis, empyema, mucocele) was decompressed.
2. Hartmann’s Pouch stones (HPS) were either pushed back into the gallbladder or occasionally removed after opening the Hartman Pouch to facilitate the dissection of the cystic plate.
3. The cystic lymph node was identified as it is a reliable marker of the underlying cystic artery.
4. The presence of the duodenum in the view of the operative field was considered a risk factor for BDI and a new target area was chosen further laterally.
5. A thick-walled gallbladder could be adherent to the duodenum or the lateral wall of the bile duct so subserosal dissection was preferred (Fig. 1).
6. A contracted gallbladder (e.g. contracted fundus has caused notching of the liver edge) may suggest the common bile duct being drawn laterally (Fig. 2). Dissection around the body of the gallbladder or fundus first dissection (FFD) was considered.
7. If CVS could not be obtained, a transvesical IOC through the body or infundibulum of the gallbladder was performed (Fig. 3).

**Fig. 4:**
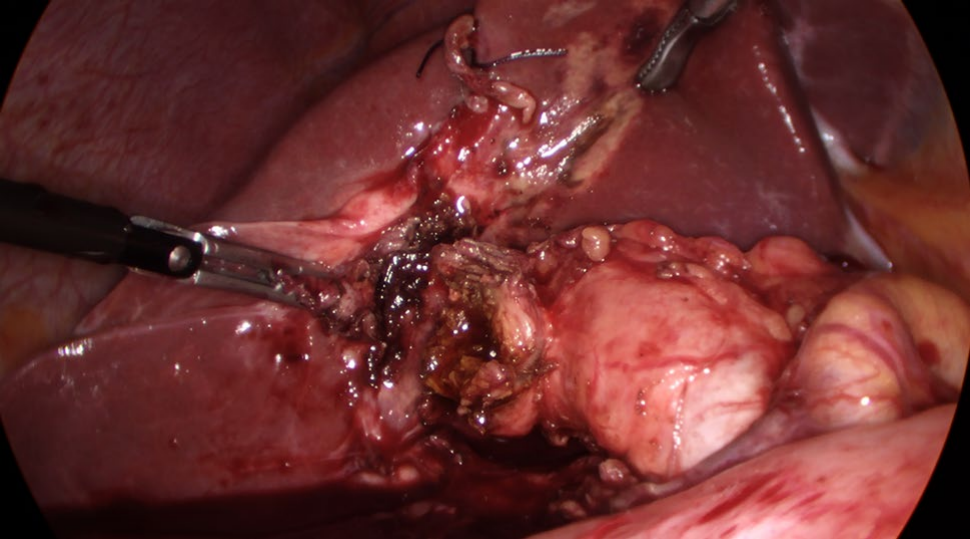
Following limited funds first dissection, the "funnel" technique is used to access the posterior aspect of the Hartman's Pouch allowing the creation of a stump

If IOC could not be obtained, gallbladder body was divided horizontally creating a “funnel-shaped remnant” with the whole contour of the Hartman’s pouch becoming visible, allowing safe blunt posterior dissection (Fig. 4). In our practice this replaces subtotal cholecystectomy which was not performed during this series.

### Statistical analysis

Categorical variables are expressed as *n* and percentage (%) and continuous as mean ± standard deviation. For comparison between CVS and no-CVS groups differences were assessed with Student *T* test for continuous variables and Chi-square or Fisher Exact test for categorical variables. Multivariable analysis was also performed for CVS and no-CVS cohorts. *p* value of < 0.05 was considered statistically significant. All analyses were performed using IBM SPSS 22.

## Results

1060 consecutive LC were performed over four years. The median age was 53 years and the male to female ratio was 1:2.1. The majority were emergency admissions (54.9%). The primary admission diagnosis was simple biliary colic in 43.6%, prolonged biliary colic in 16.8%, obstructive jaundice with or without cholangitis in 18.8%, acute cholecystitis in 11.8% and acute pancreatitis in 8.6%. One hundred and sixty patients (15.1%) had previous biliary emergency admissions, the majority (*n* = 123) by other departments or hospitals. Only 37 patients (3.5%) were previously admitted under the care of the biliary team but had not undergone index admission surgery.

The CVS could not be displayed in 15.8%: 55.6% with difficulty grade IV and 92.3% with difficulty grade V. Four in ten LC were considered difficult (grades III–V). IOC was attempted in all patients and was successful in 98%. IOC could not be done in 21 patients: CVS was achieved in 16 and could not be achieved in 5.

Preoperative criteria associated with a failure to achieve the CVS included age over 60, male sex, emergency admission, past or current acute cholecystitis and previous biliary interventions (Table 1). Operative factors predictive of failed CVS will not surprise surgeons (Table 2): adhesions to duodenum or colon, accessory cystic artery, gallbladder condition other than chronic cholecystitis and cholecystoduodeno/colic fistula. There were no bile duct injuries or open conversions in either group. The mean number of hospital episodes in this series, including previous and re-admissions, was 1.2 per patient. As would be expected there were significant differences in the median duration of surgery and the median hospital stay, both being longer in cases where the CVS could not be achieved. On the other hand, although the overall morbidity rate was higher in cases where displaying the CVS was not possible (Table 3), there were no differences in each Clavien- Dindo class between the two groups (Table 4) probably due to the small numbers. Most of the significant perioperative complications occurred in patients who underwent bile duct explorations. This included six of seven postcholecystectomy bile leaks (0.6%), three of whom had failed CVS (Table 5).

**Table 2:** Predictive preoperative criteria comparing CVS vs. No CVS

Characteristics	CVS established *n* = 893 (84.2%)	No CVS established *n* = 167(15.8%)	*p* value	OR (95% CI)
Age > 60 years (*n* = 355)	268 (75.5%)	87 (24.5%)	< 0.001	0.39 (0.28–0.55)
Male sex (*n* = 339)	256 (75.5%)	83 (24.5%)	< 0.001	0.41 (0.29–0.57)
Emergency admission (*n* = 582)	462 (79.4%)	120 (20.6%)	< 0.001	0.42 (0.29–0.60)
Timing of surgery in days				
0–1 (*n* = 748)	641 (85.7%)	107 (14.3%)	REF	REF
2–5 (*n* = 200)	163 (81.5%)	37 (18.5%)	0.142	0.74 (0.49–1.11)
≥ 6 (*n* = 112)	89 (79.5%)	23 (20.5%)	0.086	0.65 (0.39–1.07)
Admission diagnosis^a^				
Acute Biliary colic (*n* = 179)	168 (93.9%)	11 (6.1%)	REF	REF
Chronic biliary colic (*n* = 463)	418 (90.3%)	45 (9.7%)	0.15	0.61 (0.31–1.20)
Obstructive jaundice (*n* = 167)	135 (80.8%)	32 (19.2%)	< 0.001	0.28 (0.13–0.57)
Acute pancreatitis (*n* = 92)	84 (91.3%)	8 (8.7%)	0.436	0.69 (0.27–1.77)
Acute cholangitis (*n* = 33)	25 (75.8%)	8 (24.2%)	< 0.001	0.20 (0.08–0.56)
Acute cholecystitis (*n* = 126)	63 (50.0%)	63 (50.0%)	< 0.001	0.07 (0.03–0.13)
Previous admission (*n* = 150)	105 (70.0%)	45 (30.0%)	< 0.001	0.36 (0.24 – 0.54)
Previous biliary surgery (*n* = 6)^b^	2 (33.3%)	4 (66.6%)	< 0.001	0.09 (0.02–0.50)
Previous ERCP (*n* = 13)	5(38.5%)	8 (61.5%)	< 0.001	0.11 (0.04–0.35)

*REF* Reference Group

^a^More than one characteristic entered occasionally

^b^Previous biliary surgery includes: cholecystostomy, cholecystectomy, common bile duct exploration

**Table 3:** Intraoperative findings in cases with CVS vs No CVS

Characteristics	CVS established *n* = 893(84.2%)	No CVS established *n* = 167 (15.8%)	*p* value	OR (95% CI)
Operative difficulty grade				
I (*n* = 353)	353 (100%)	0	REF	REF^b^
II (*n* = 282)	279 (98.9%)	3 (1.1%)	0.087	REF^b^
III (*n* = 216)	185 (85.6%)	31 (14.4%)	** < 0.001**	0.03 (0.01–0.09)
IV (*n* = 153)	68 (44.4%)	85 (55.6%)	** < 0.001**	0 (0–0.01)
V (*n* = 52)	4 (7.7%)	48 (92.3%)	** < 0.001**	0 (0–0)
No record (*n* = 4)	4 (100%)	0	1	N/A
Adhesions to gallbladder and duodenum (*n* = 515)	377 (73.2%)	138 (26.8%)	** < 0.001**	0.15 (0.10–0.23)
Adhesions to gallbladder, duodenum and hepatic flexure (*n* = 212)	100 (47.6%)	110 (52.4%)	** < 0.001**	0.07 (0.04–0.10)
Calot’s triangle abnormal (*n* = 140)	46 (32.9%)	94 (67.1%)	** < 0.001**	0.04 (0.03–0.06)
Accessory cystic artery (*n* = 332)	239 (72.0%)	93 (28.0%)	** < 0.001**	0.29 (0.21–0.41)
Gallbladder condition^a^				
Chronic cholecystitis (*n* = 721)	693 (96.1%)	28 (3.9%)	REF	REF
Hartmann’s pouch stone (*n* = 175)	105 (60.0%)	70 (40.0%)	** < 0.001**	0.06 (0.04–0.10)
Contracted (*n* = 119)	62 (52.1%)	57 (47.9%)	** < 0.001**	0.04 (0.03–0.07)
Empyema (*n* = 99)	39 (39.4%)	60 (60.6%)	** < 0.001**	0.03 (0.02–0.05)
Acute cholecystitis (*n* = 60)	43 (71.7%)	17 (28.3%)	** < 0.001**	0.10 (0.05–0.20)
Mucocele (*n* = 41)	33 (80.5%)	8 (19.5%)	** < 0.001**	0.17 (0.07–0.39)
No record (*n* = 24)	22 (91.7%)	2 (8.3%)	0.275	0.44 (0.10–1.98)
Mirizzi syndrome (*n* = 14)	3 (21.4%)	11 (78.6%)	** < 0.001**	0.05 (0.01–0.17)
Cholecysto-duodenal/cholecysto-colic fistula (*n* = 9)	2 (22.2%)	7 (77.7%)	** < 0.001**	0.18 (0.06–0.52)

Significant *p* values are in bold

^a^More than one characteristic entered occasionally

^b^Grade I and II combined as denominator 0

**Table 4:** Operative and postoperative outcomes comparing CVS and No CVS

Characteristics	CVS established *n* = 893	No CVS established *n* = 167	*p* value	OR (95% CI)
Fundus first dissection (*n* = 50)	5 (10.0%)	45 (90.0%)	< 0.001	0.02 (0.01–0.04)
Conversion to open/bile duct injury (*n* = 0)	0	0	1	N/A
Duration of surgery, median (range)	50 (22–325) min	95 (38–390) min	< 0.001	N/A
Duration of hospital stay, median (range)	4 days (1–60)	9 days (1–46)	< 0.001	N/A
Perioperative complication (*n* = 70)	49 (70.0%)	21 (30.0%)	< 0.001	0.40 (0.24–0.69)
Mortality	1 (50.0%)	1 (50.0%)	0.183	0.19 (0.01–2.99)

**Table 5:** Clavien–Dindo Grade complications comparing CVS and No CVS

Clavien-Dindo Grade	Nr (% patients)	CVS established *n* = 893	No CVS established *n* = 167	*p* value	OR (95% CI)
1	41 (3.9%)	32	9	0.267	REF
2	14 (1.3%)	9	5	0.039	0.51 (0.14–1.89)
3a	9 (0.8%)	5	4	0.018	0.35 (0.08–1.59)
3b	4 (0.4%)	2	2	0.060	0.28 (0.03–2.28)
5	2 (0.2%)	1 (50.0%)	1 (50.0%)	0.183	0.28 (0.02–4.95)
Total	70				

The only dissection-related complications, where the CVS could not be displayed, were an inadvertent opening of a chole-cystoduodenal attachment (suture repair with no consequences) and the disconnection and repair of a confirmed cholecystocolic fistula. The patient developed a postoperative collection requiring percutaneous drainage followed by re-laparoscopy and temporary ileostomy.

ERCP and stenting was necessary in one patient with Mirizzi Syndrome Type III who had a bile leak. This was followed by a laparotomy and bilioenteric anastomosis. Another patient had a slow postcholecystectomy bile leak requiring percutaneous drainage of a collection followed by ERCP and stenting and re-laparoscopy for washout.

Two deaths occurred during this study. One patient developed mesenteric ischaemia and total bowel infarction was found at laparotomy after an uneventful LC for a perforated gallbladder. Another patient died of pneumonia three weeks after bile duct exploration for Mirizzi Type II.

## Discussion

The Critical View of Safety could not be established in 1 out of 6 consecutive laparoscopic cholecystectomies in this prospective single surgeon dataset of over 1000 operations. With increasing operative difficulty, based on the Nassar Scale [11], the ability to demonstrate CVS progressively fell from 100% (grade I) to 7.7% (grade V; *p* < 0.001). All pathological states of the gallbladder were associated with a lower rate of being able to demonstrate the CVS when compared to the floppy gallbladder (“chronic cholecystitis”).

There were no bile duct injuries in this series despite this inability to uniformly achieve CVS. Although the grade of operating surgeon was documented, this was not evaluated as the senior surgeon eventually determined the failure of obtaining the CVS and carried out any alternate/salvage strategy. Others have also reported no BDI despite not displaying CVS: Avegerinos et al. where CVS was possible in 95.4% of 1046 LC [13] and Sanjay P et al. where CVS was obtained in 87% of 447 LC [14]. A Dutch study found video confirmation of CVS in only 18.7% of LC where this was recorded as reached in the operation note [15] Although undoubtedly a useful tool of target identification during LC, the introduction of the CVS has had no effect on the incidence of bile duct injury–which Strasberg attributes to a poor understanding of the criteria for CVS [6].

It would seem unlikely that surgeons not understanding this simple concept or that the lack of photographic documentation of the CVS is to blame for biliary injuries. A multi-society consensus conference on prevention of bile duct injury during cholecystectomy suggested that no direct comparative evidence was identified to support the critical view of safety (CVS) over other methods for anatomic identification [16]. It concluded that there is no substantial evidence that reasonable efforts to achieve the CVS have been associated with undesirable effects. The conference quoted only one report of bile duct injury occurring during attempts to achieve the CVS [17]. However, it is important to point out the CVS is a conclusion of the dissection process. It would be unusual that injuries of the main bile ducts would occur after displaying the CVS, although the descriptions of CVS do not focus on how to achieve this end point.

The current study suggests that as it becomes more crucial to achieve all 3 CVS components, it actually becomes much more difficult to do so. Inability to display a CVS was associated with a higher operative difficulty grade (Grade IV and V) and a higher morbidity rate in this study. It may be argued therefore that operative difficulty grading is a more accurate predictor of CVS failure. 79.6% of failed CVS occurred in difficulty Grades IV and V LC. A high difficulty grade could be an early warning that displaying a CVS is unlikely. Although it is possible to continue attempting to display the anatomy, alternate approaches could be considered and implemented e.g. intraoperative time-out via Doublet View or multiple techniques including “bail out” strategies as advocated by SAGES [8]. In our view determining difficulty grading, predicting the potential failure of displaying the CVS and the early adoption of alternate strategies is a safer approach than relying on the subjective judgement of “approaching significant risk” or the recognition that conditions were becoming “too dangerous” used by SAGES.

**Fig. 5:**
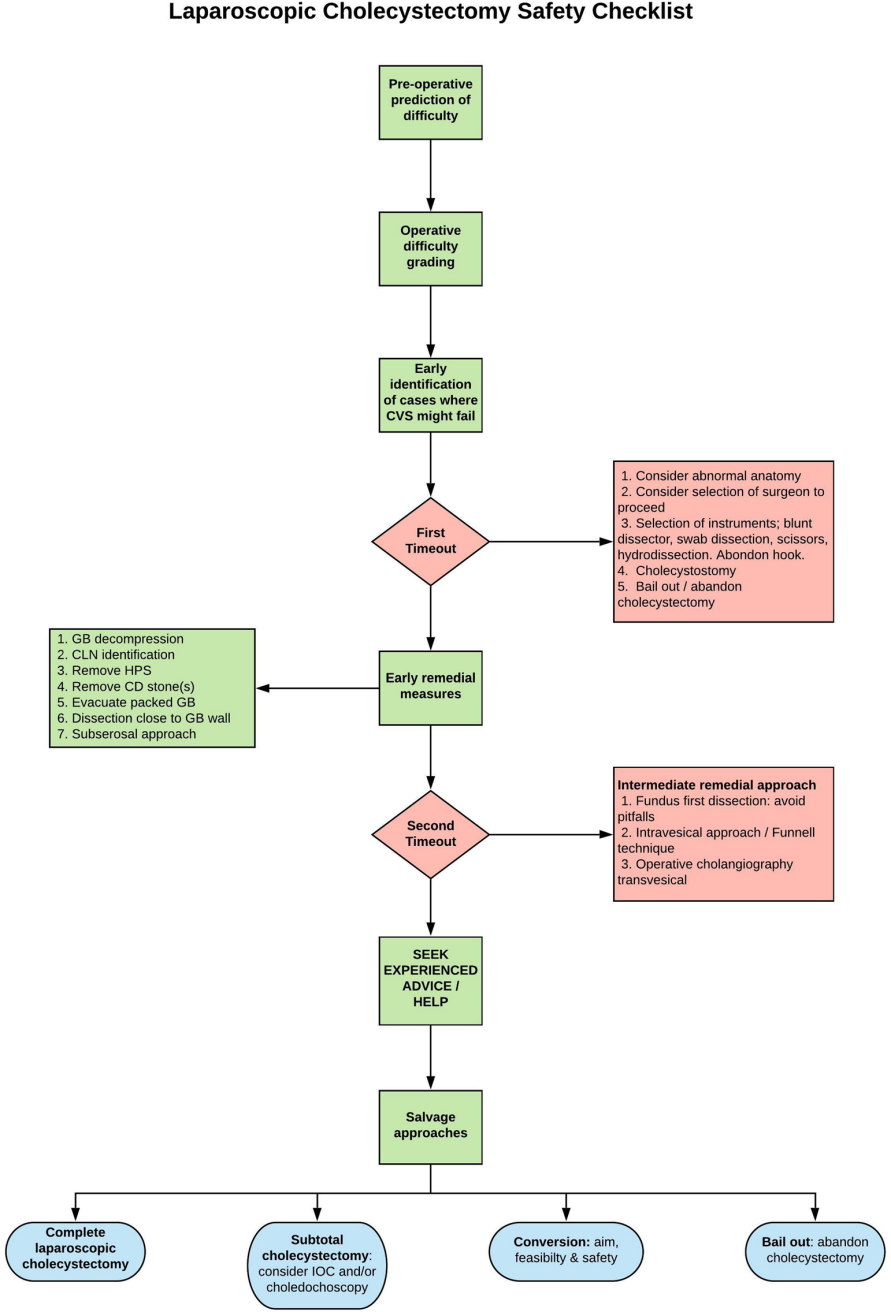
Safety pathway, time-out and salvage strategies for laparoscopic cholecystectomies where failure of CVS is predicted

In our practice, we use the term “salvage technique”, aiming to complete the cholecystectomy without bile duct injury (Fig. 5) while ensuring an optimal patient outcome: no stones in the gallbladder remnant/cystic duct and no postcholecystectomy bile leak. It must be appreciated that “bailing out” can be due to inexperience at recognising what is “too dangerous” or the reluctance to use alternative safety approaches or to seek advice from a colleague. Laparoscopic cholecystostomy is a difficult choice to make (which needs to be made early) but may be the safest bail out strategy [18]. Van de Graaf et al. [19] reported a systemic review of bile duct injury prevention in which 7 articles covered laparoscopic subtotal cholecystectomy (LSC). A median of 9.1% (IQR 6.3–10.3%) rate of LSC was reported with a median bile leak rate of 6.3% (IQR 0.85%-12.5%). The infundibular technique may be considered—Vettoretto found no difference in morbidity when comparing CVS to infundibular technique but due to insufficient power, the comparison was inconclusive [20]. Fundus first dissection (FFD) has been shown by Cengiz et al. [21] to result in a reduced bile duct injury rate of 0.07% compared to 0.9% with the conventional approach. An important component of the SAGES Universal Culture of Safety initiative is the “liberal use of cholangiography”[8].

No objective definition of difficulty was used in any studies addressing the CVS and no mention was made of any of the reported difficulty grading classifications or scores. Surgeons vary in their level of experience and skill and, under adverse operative conditions, may fail to adequately judge the complexity of the pathology they encounter. A structured definition of difficulty may help a surgeon reach an early sound assessment. In our study we used a difficulty grading system published by the senior author in 1995 to grade every cholecystectomy at the point of commencement of pedicle dissection or soon after, when specific criteria were identified. This allows for objective assessment based on a descriptive classification into five difficulty grades. It is subsequently possible to make the decisions described in the SAGES guidance, decide who/how/when to continue with the dissection and what salvage approach is to be adopted.

The availability of methods to facilitate intraoperative imaging of the biliary tree e.g. laparoscopic ultrasound (LUS), visual fluorescent cholangiography using Indocyanine green (ICG) and improved visualisation of the anatomy using 3D technology can add to the surgeon’s ability to better identify the biliary anatomy and would no doubt enhance the safety of LC. LUS has been reported to ensure a safe plane of dissection and to avoid biliary and vascular complications particularly in the presence of anatomical anomalies. Sebastian et al. advocated LUS as a non-invasive technique which, unlike cholangiography, can be used anytime, does not involve cystic duct cannulation and does not use contrast or X-Ray [22]. Although their study excluded acute cholecystitis it had the relatively high conversion rate of 4.8%. However, LUS has a decreased sensitivity for detecting stones in the retroduodenal bile duct and for identifying abnormal ductal anatomy, when compared to cholangiography. The unavailability of the equipment and the expertise for the interpretation of the images may be added limitations in many departments.

ICG fluorescent cholangiography is non-invasive and guides safe dissection through real-time identification of biliary structures. Pesce et al. [23] conducted a systematic review concluding that ICG is highly sensitive for the detection of important biliary anatomy namely the cystic duct, common hepatic duct and common bile duct before commencing the dissection of the hepatocystic triangle and can thus help to prevent bile duct injuries. However, cystic duct and common bile duct visualisation were reduced in patients with a BMI higher than 35. Although Bleszynski et al. reported singular biliary structure detection rates of 90%, 84% and 48% for the cystic duct, common bile duct and common hepatic duct with ICG, the cumulative biliary structures ICG visualisation rates were much lower [24]. However, the authors admitted to selection bias as they did not include any emergency cholecystectomies which would obviously be expected to pose difficulty in the presence of various degrees of inflammation. Direct injection of ICG into the gallbladder has also been described, requiring no learning curve, and no complications have been reported [25]. 3D laparoascopy was reported to shorten operative time and enhance depth perception in five randomised studies analysed for a systematic review [26]. However, a recent randomised trial by Schwab et al. [27] concluded that 3D laparoscopy did not reduce overall operative time or error frequency in LC performed by specialist surgeons. In agreement with our findings, they observed that case difficulty, according to the Nassar Scale, was a major confounding variable with a larger impact on operative time than the imaging system used.

It is interesting that no studies addressing the CVS and alternate strategies considered the role of dissection instruments in bile duct injuries, given that the diathermy hook and endoclips are the main variables between open and laparoscopic cholecystectomy. Some authors discussed hydrodissection combined with blunt dissection, using the suction probe, to explore a difficult pedicle [28, 29]. In our study the diathermy hook had no role in dissection at any stage during LC. Studying the identification and categorisation of technical errors, Tang et al. [30] conducted an Observational Clinical Human Reliability Assessment (OCHRA) during 200 LC. More errors and a higher error probability were noted with the use of the electrosurgical hook when compared to dissection graspers. Failure to visualise the tip of the hook was causative in 68% of the errors. The use of the hook resulted in more “consequential” errors and serious injuries. Applying excessive force and wrong instrument direction/spatial orientation resulted in 53% and 42% of the errors committed using the hook. They concluded that “the poor design of the electrosurgical hook knife is largely responsible for the error modes “. The diathermy hook is the most commonly used dissection instrument and while it may be safely used in most circumstances by most surgeons it may not be the optimal instrument to use where the anatomy is not clear or dense fibrosis is encountered e.g. contracted gall bladders or Mirizzi Syndrome. Randomised studies are required to compare the safety of different laparoscopic instruments used during LC and to identify risk factors associated with them.

As may be expected, this study showed that failure to display a CVS was associated with a higher operative difficulty grade (Grade IV and V) and a higher overall morbidity rate. However, most of the significant perioperative complications occurred in patients who underwent bile duct explorations and were unrelated to whether or not CVS was achieved.

The adoption of alternate dissection strategies and IOC in this study has resulted in the optimisation of important operative outcome parameters including less reliance on subtotal cholecystectomy and cholecystostomy, avoiding their potential adverse consequences. We also report safe laparoscopic completion of complex cases with Mirizzi Syndrome [31], cholecysto-duodenal and cholecysto-colic fistulae while avoiding open conversion and bile duct injuries.

## Conclusion

The CVS is the end product of a process of dissection and bile duct injuries occur before the conclusion of that process. While the CVS is achievable in the majority of LCs, obtaining CVS will not be possible with higher degrees of difficulty. A complete strategy of safety should therefore include early recognition of difficulty and identification of cholecystectomies where the CVS will be impossible to display in order to guide the utilisation of new intraoperative technologies to clarify the anatomy and the selection of appropriate instruments and dissection approaches. Whether to complete the cholecystectomy or use a salvage strategy will depend on the judgement and skills of the operating surgeon.
